# Microsatellite-Based Genetic Characterization of the Indigenous Katjang Goat in Peninsular Malaysia

**DOI:** 10.3390/ani11051328

**Published:** 2021-05-07

**Authors:** Mohd Adhan Ernie Muneerah, Nur Aida Md Tamrin, Mohd Shahrom Salisi, Shahrizim Zulkifly, Siti Shaidatul Maisarah Ghazali, Jackson Jenun Temuli, Mohd Hifzan Rosali, Shariffah Nazari, Wan Mohd Kamil Wan Nik, Kamalludin Mamat-Hamidi

**Affiliations:** 1 Institute of Tropical Agriculture and Food Security, Universiti Putra Malaysia, Serdang 43400, Selangor, Malaysia; ernie@dvs.gov.my; 2 Department of Veterinary Services, Ministry of Agriculture and Food Industries, Precinct 4, Federal Government Administration Centre, Putrajaya 62630, Malaysia; shariffahnazari@dvs.gov.my (S.N.); wankamil@dvs.gov.my (W.M.K.W.N.); 3 Department of Animal Science, Faculty of Agriculture, Universiti Putra Malaysia, Serdang 43400, Selangor, Malaysia; nuraida.mtamrin@upm.edu.my (N.A.M.T.); shaidatulmaisarah45@gmail.com (S.S.M.G.); jacksonjenun95@gmail.com (J.J.T.); 4 Department of Veterinary Preclinical Sciences, Faculty of Veterinary Medicine, Universiti Putra Malaysia, Serdang 43400, Selangor, Malaysia; shahrom@upm.edu.my; 5 Department of Biology, Faculty of Science, Universiti Putra Malaysia, Serdang 43400, Selangor, Malaysia; shahrizim@upm.edu.my; 6 Animal Science Research Centre, Malaysian Agricultural Research and Development Institute, Ministry of Agriculture and Food Industries, Persiaran MARDI-UPM, Serdang 43400, Selangor, Malaysia; hifzan@mardi.gov.my

**Keywords:** genetic diversity, population genetic relationship, population genetic structure, network analysis

## Abstract

**Simple Summary:**

Genetic characterization is one of the tools to assess the genetic diversity of livestock breeds towards the goals of conservation and sustainable use. This research aimed to assess the genetic diversity, population, genetic relationship, and structure of the Malaysian indigenous Katjang goat breed, which has been reported to be at risk of extinction by the Food and Agriculture Organization of the United Nations (FAO). Through assessment from microsatellite DNA markers, this breed was found to have low genetic diversity and showed evidence of high inbreeding. This breed might also have undergone population bottlenecks in the past. Through combined data analysis with other breeds and populations, available through data from published research, the Katjang goat population was found to have interconnection and form the centre of the network; it was also found to be the centroid of the multidimensional scaling plot. The findings of this research help in the understanding of the current genetic diversity of this breed and the need for its conservation.

**Abstract:**

The Katjang goat is the only indigenous domestic goat breed in Malaysia. Following a national baseline survey from 2001 to 2002, this breed was reported to the FAO as being at risk of extinction. In this study, 36 microsatellite markers were screened, and 25 polymorphic markers were used to analyze the genetic structure of the Katjang goat breed in Peninsular Malaysia. A sample set of data derived from another 10 populations from three published research studies was used as an outgroup for an inter-population genetic study. The analysis showed that the mean value of the observed heterozygosity was 0.29 ± 0.14, and the expected heterozygosity was 0.72 ± 0.14, which indicated low genetic diversity. The inbreeding coefficient, F_IS_, was high, at 0.46. Significant (*p* < 0.01) deviations from the Hardy Weinberg equilibrium were noted for all loci. The bottleneck analysis using the Wilcoxon Rank test under the two-phase model of mutation was significant (*p* < 0.01) for heterozygosity excess, which suggested that the Katjang breed had undergone significant population reduction in the past. Through combined analysis of data from publicly available research, almost the entire population of Katjang goats represent the centroid and are grouped together on a multidimensional scaling plot, except for the Terengganu population. Network analysis revealed that the goat population from Pahang formed the centrality of the network.

## 1. Introduction

In Malaysia, the total goat population was estimated to be 312,571 heads in the year 2019 [1]. The production of mutton was 4200.6 metric tons, while the demand was 35,489.8 metric tons; this meant the self-sufficiency level for mutton was only 11.84% [1]. As a result, Malaysia imported 10,224 live goats and 31,348.7 metric tons of mutton to cater to the demand [2].

Goat breeds in Malaysia can be classified as indigenous, crossbreed, and introduced or imported breeds. Among all the domesticated goat breeds available in Malaysia, the Katjang goat is the only indigenous breed [3]. This breed is morphologically similar to Indian local goats, probably due to migration during ancient trade routes from India to Southeast Asia [3]; the breed is also very similar to goats from the Philippines, Taiwan, southwest Japan and the South China [4]. This breed also might have entered Peninsular Malaysia through India [5] and east of Malaysia via China [6].

**Figure 1:**
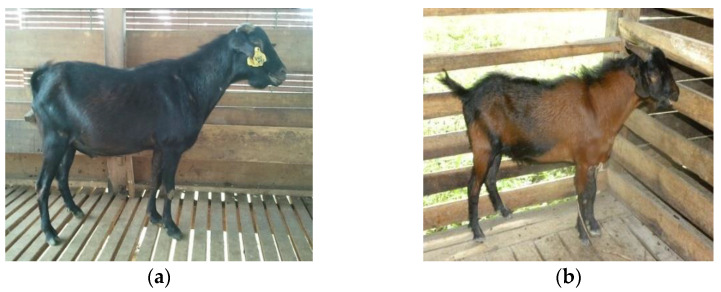
Indigenous Katjang goat of Malaysia. (**a**) Adult (male) Katjang goat with black coat colour; (**b**) adult (male) Katjang goat with black and dark brown coat colour.

The Katjang goat is also called the Kacang (in the Malay language) or pea and bean goat due to its small size [7] and preference for eating bean leaves [8]. The goats can only be found in small pockets in several parts of Malaysia [9]. The indigenous Katjang goat breed was documented as black in colour sometimes with white patches at the centre of the body, under the belly and on the face [3,10,11](Figure 1a). A white colour belt or Lakenfeld pattern is also occasionally found [12]. There are also goats that are dark brown coloured, with black at the head and a black stripe around the middle of the body or at the tail and feet [5,13](Figure 1b). They are prolific, and twinning is common [14]. According to Peters et al. [15], the milk yield is low and utilized entirely for kid rearing.

The Katjang goat is a hardy animal that can utilize a wide range of vegetation. It is better suited to the rich bush and tree growth of the wet tropics. It can also survive on the very poor browse of the secondary jungle, on scrub, and by scavenging the village area [16]. As an indigenous goat, it adapts well to the local environment and has more tolerance to the heat and ticks experienced in the local climate [11]. Despite being hardy, its growth potential is relatively poor [17]. Its size is smaller than the imported goat breeds in Malaysia, making it less favoured than the larger-sized breeds. The male Katjang has an average height at withers of 60–65 cm and the female has a height of 56 cm [14,18]. Mature males and females weigh approximately 25 kg and 20 kg, respectively [11]. Other imported breeds in Malaysia, namely Boer and Red Kalahari breeds, are reported have an average height of 68.12 cm and 74.16 cm, respectively, for females. The weight of a female Boer is 58.23 kg, and the Red Kalahari has a weight of 52.19 kg [19]. Therefore, crossbreeding by random mating of the Katjang breed with various imported breeds occurs widely to improve their productivity, especially to improve body weight [20,21]. The practice is very successful to the extent that the number of pure Katjang goats in Malaysia is difficult to determine [22]. This is a horrific situation for the Katjang goat, whose genetic purity and diversity should be preserved as a national asset.

During a national baseline survey of livestock breeds from 2001 to 2002, the Katjang goat breed in Malaysia was classified as being at risk for extinction [9] and given unknown status in the Domestic Animal Diversity Information System under the FAO [23]. According to the FAO [24], proper actions need to be taken for animals with unknown status, since the breed could be critical, endangered and vulnerable. In 2013, Malaysia published the Livestock Breeding Policy, which suggested that the Katjang goat should be conserved in situ and ex situ, and that the breed should be improved [22]. In view of these, assessment of molecular genetic information can be utilized as one of the keys in the management of sustainable conservation and improvement strategies. 

In this study, the genetic characterization of the Katjang goat was done using DNA microsatellite markers. Microsatellite markers were chosen due to their high variability, co-dominant inheritance, and relative ease of detection, making them very useful for detecting differences among populations and between individuals [25]. Microsatellites also do not encode proteins and are thus assumed to be selectively neutral [26]. Their occurrence in protein coding regions are relatively rare [27].

To date, genetic characterization of the Katjang goat in Malaysia has been reported using protein loci for West Malaysia (Sabah and Sarawak) populations [6]. Genetic diversity through heterozygosity was also reported for Katjang goats in Malaysia using microsatellite markers in 2013 [28].The objective of the present study was to undertake microsatellite-based characterization of the Katjang goat through evaluation of genetic diversity, the inbreeding coefficient and bottleneck analysis as well as their population structure and network analyses.

## 2. Materials and Methods

**Figure 2:**
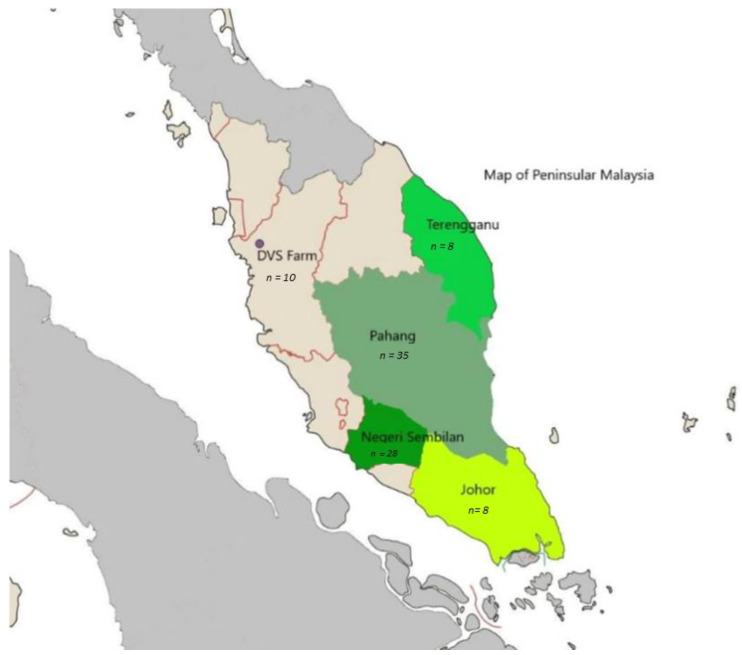
Geographical locations of the origins of the 89 sampled Katjang goats from Peninsular Malaysia, grouped by states (represented by different shades of colours). Of these, 79 samples were from various farms in four different states in Malaysia and 10 samples were from a government farm (DVS Farm) in the state of Perak.

The experimental conditions in the present study were approved by the Institutional Animal Care and Use Committee of Universiti Putra Malaysia, Malaysia (AUP no.: RO54/2018). A total of 79 purebred Katjang goats were randomly sampled from various farms of four different states in peninsular Malaysia. The purebred individuals were randomly selected based on their phenotypic features, as described by historical data [3,5,10,11,13]. The samples collected were from Negeri Sembilan (*n* = 28), Pahang (*n* = 35), Johor (*n* = 8) and Terengganu (*n* = 8) (Figure 2). Since there was indiscriminate practice of crossbreeding on this breed, efforts were made to select unrelated purebred animals to minimize the degree of relationship; thus, some populations have small number of samples. A further 10 purebred Katjang goats were sampled from the Department of Veterinary Services Malaysia farm (DVS Farm) in Pondok Tanjung, Perak (Decimal degrees: 5.031, 100.731). The original Katjang goats in this farm were obtained from various places in peninsular Malaysia and had inbred with one another since 2010. All blood samples were collected from the jugular vein of each animal using EDTA-coated tube.

DNA was extracted from the whole blood using the DNEasy Blood & Tissue Kit (QIAGEN, Hilden, Germany). The concentration and purity of the genomic DNA were analyzed using a Biophotometer (Eppendorf, Harmburg, Germany), and the quality of the genomic DNA was tested using gel electrophoresis. A total of 36 microsatellite markers were used in this study. Thirty microsatellite loci were based on the list recommended by ISAG & FAO’s Domestic Animal Diversity Information System-Measurement of Domestic Animal Diversity [26], and another six microsatellite markers were selected from other goat studies [6,29,30,31].

DNA amplifications were done using Polymerase Chain Reaction (PCR), with optimized protocol for each locus. PCR was carried out on 50 ng/µL genomic DNA in a 50 µL reaction volume using primers, template DNA (50 ng/µL), double distilled water and reagents (Promega, Madison, WI, USA) consisting of 200 mM dNTP, 1 unit of Taq DNA Polymerase, 1.5 mM of MgCl_2_, and 1X polymerase buffer. PCR was done in a thermal cycler (MJ Research, Waltham, MA, USA). DNA amplifications were set up with the initial denaturation at 94 °C for 5 min, followed by 35 cycles of denaturation (94 °C for 30 s), annealing (53–67 °C for 30 s) and elongation (72 °C for 30 s), followed by a final extension of 72 °C for 3 min. 

Microsatellite genotypings were done using the MetaPhor agarose (Lonza, Morriston, NJ, USA) gel electrophoresis technique with 1X Tris borate EDTA (TBE) buffer (Promega, Madison, WI, USA). Electrophoresis was conducted at 70 V for two hours. Alleles were observed under ultraviolet (UV) light using the Gel Documentation System (Vilber Lourmat, Collegien, France). Allele sizes generated from the microsatellite markers were estimated in comparison to 25bp DNA ladder (Promega, Madison, WI, USA) using the Gel Analyzer software, version 2010a [32]. The sizes of each of the alleles were recorded in the Microsoft Excel (version 2103, Microsoft Corporation, Redmond, WA, USA) format.

The CONVERT software, version 1.31 [33], was used to convert the matrix data from Microsoft Excel into the format required by the statistical software. Statistical analyses were performed using Popgene version 1.32 [34] for observed and effective allele numbers [35] for overall Katjang breed across microsatellite loci. The software was also used to generate observed and effective heterozygosities according to Levene [36] as well as Hardy Weinberg equilibrium (HWE) test [37] and inbreeding coefficient (F_IS_) according to Nei [38] for overall breed across microsatellite loci and within each five different populations. The Bottleneck software, version 1.2.02 [39], was used to test the bottleneck hypothesis under a two-phase model of mutation (TPM) [40] and one-tail heterozygosity excess of Wilcoxon rank test [41]. Mode-shift distortion using allele frequency data was also used to detect recent occurrences of bottleneck [42].

For inter-population genetic relationships and structure of the five populations, the data were analyzed by combining allele frequency data of other breeds and populations from publicly available data repositories and literature [43,44,45] to be used as outgroups. The data was filtered for shared microsatellite loci for combined analysis. From the combined data, 10 populations were selected as specified in Table 1. Eleven shared microsatellite loci were used as specified in Table 2. For genotype data, CONVERT software, version 1.31 [33], was used to convert the data into allele frequency data. The allele frequency data was then used to generate Unweighted Pair Group Method with Arithmetic Averaging (UPGMA) tree clustering from D_A_ genetic distance [46] using POPTREE2 software [47] with the bootstrap value of 1000. GenoCline version 1.5 [48] was used to generate multi-dimensional scaling (MDS) [49] for population structure based on Reynold’s F_ST_ values [50]. EDENetwork, version 2.18 [51], was used to create a network analysis between populations based on the Goldstein’s genetic distance [52]. The networks were built based on the percolation threshold level generated automatically by the software to identify strongly clustered genotypes [53]. 

## 3. Results

### 3.1. Microsatellite Marker Assessments

From the 36 microsatellite markers examined, one microsatellite locus (BM6444), which was suggested by the ISAG & FAO’s Domestic Animal Diversity Information System-Measurement of Domestic Animal Diversity [26], failed to produce any allele. The absence of allele in the locus was possibly due to the presence of a null allele. Table 3 shows the allelic diversity based on 35 loci. A total of 198 alleles were detected across the 35 microsatellite loci from all the studied populations. Thirty-one microsatellite loci showed polymorphic variations, while the other four markers (INRABERN172, ILTS005, MAF209 and RM004) were monomorphic in the Katjang goats in this study.

From the thirty-one polymorphic markers, the number of alleles observed across the polymorphic loci varied between two to fifteen alleles. Another six markers (SRCRSP23, INRA023, MCM527, INRA063, ETH10 and SRCRSP7) generated low allele numbers of between two and four.

According to Barker [55], each microsatellite marker should contain at least four alleles to reduce standard error of distance estimates between populations. Therefore, the four monomorphic markers, along with another six loci (SRCRSP23, INRA023, MCM527, INRA063, ETH10 and SRCRSP7) that generated less than four alleles, were excluded for further analyses.

### 3.2. Genetic Diversity across the Studied Microsatellite Loci

Table 4 shows the genetic diversity estimates across 25 polymorphic loci. The overall mean of the observed number of alleles was 7.24 ± 2.24, while the mean effective number of alleles (Ne) was lower, at 4.20 ± 1.8. All polymorphic markers showed less effective numbers of alleles than the observed numbers. 

The values of observed heterozygosities ranged from 0.08 at locus TCRVB6 to 0.59 at the MAF065 locus. The mean number of observed heterozygosity was 0.29 ± 0.14, which was lower than the value of the effective heterozygosity, at 0.72 ± 0.14. All markers showed higher effective numbers of alleles than the observed numbers.

All loci, except the P19 (DYA) locus, showed positive values of the inbreeding coefficient, F_IS_, which indicated heterozygote deficiencies occurred in 24 loci. Overall, the mean value of F_IS_ for the Katjang breed across the polymorphic loci was 0.46, which indicated heterozygote deficiencies in this breed. All loci significantly deviated from HWE, with a significance level of *p* < 0.01.

Heterozygosity and allele frequency data were utilized for testing the possible occurrence of bottlenecks. The Wilcoxon Rank test gave the probability of 0.00171 under the two-phase model of mutation for detecting bottlenecks in this breed. The probability was significant (*p* < 0.01) for heterozygosity excess, which suggested that the Katjang breed had undergone a population bottleneck. However, the test of bottleneck using allele frequency data of the mode-shift distribution produced a normal L-shaped distribution, which suggested that the allele frequency was not significantly distorted.

### 3.3. Genetic Diversity within Katjang Populations

Genetic diversity parameters for each population are summarized in Table 5. Observed heterozygosity ranged from 0.25 ± 0.24, in the population of Terengganu, to 0.37 ± 0.23, in the population of the DVS Farm. All populations had higher expected heterozygosity than was observed. 

All populations also showed positive F_IS_ values, ranging from 0.25 to 0.56, which indicates an excess of homozygotes due to a high level of inbreeding in all populations. Significant deviation from HWE was also detected in all populations.

### 3.4. Population Genetic Relationship and Structure

**Figure 3:**
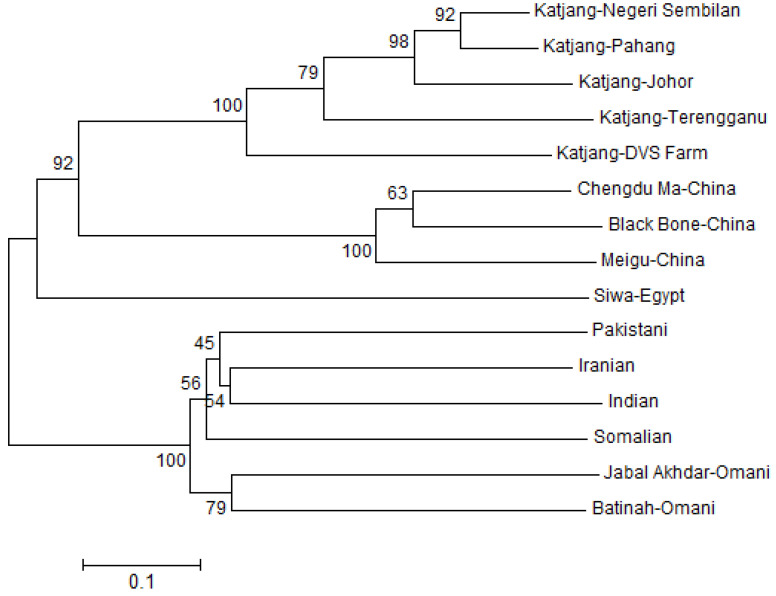
Dendogram of relationships among populations constructed using Arithmetic Averaging (UPGMA) tree clustering from D_A_ genetic distance [46]. Numbers on the nodes are percentage bootstrap values of 1000 replications.

The genetic relationship among the five populations of Katjang breed in Malaysia is represented by the dendogram tree of the D_A_ genetic distance test [46] (Figure 3). Notably, Katjang from Negeri Sembilan and Pahang are grouped together, and they are then first clustered with the Johor population, then the Terengganu population and later with the DVS Farm. All Katjang populations are then grouped with the population from China and then with the goats from Siwa, Egypt. All other populations are grouped together in another clade.

**Figure 4:**
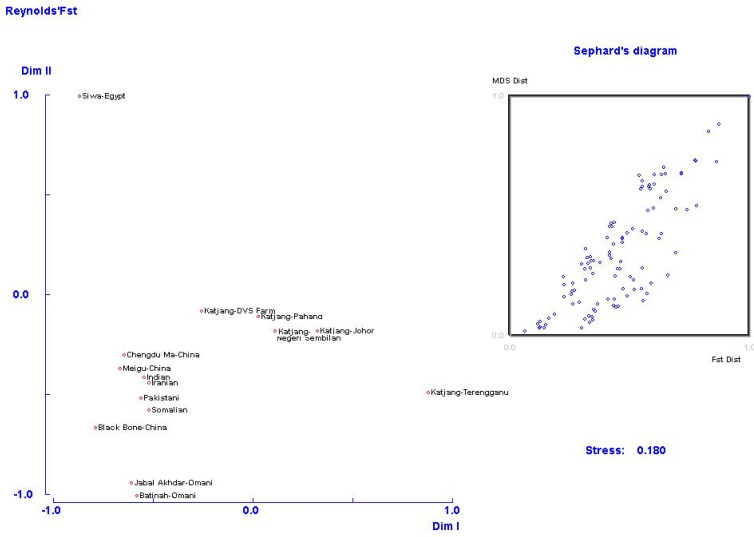
Multidimensional scaling (MDS) plot based on Reynold’s F_ST_ values [50] between all populations (stress value = 0.18).

A multidimensional scaling (MDS) plot based on Reynold’s FST values [50] was used to explore population genetic structure (Figure 4). Almost all populations of Katjang goats are grouped together and located at the centre of the MDS plot, with the exception of the Terengganu population, which is assigned to the bottom right corner. The Pahang population forms the centroid of the MDS plot. Goats from Siwa, Egypt are assigned the top left corner. All other populations are assigned the bottom left corner.

**Figure 5:**
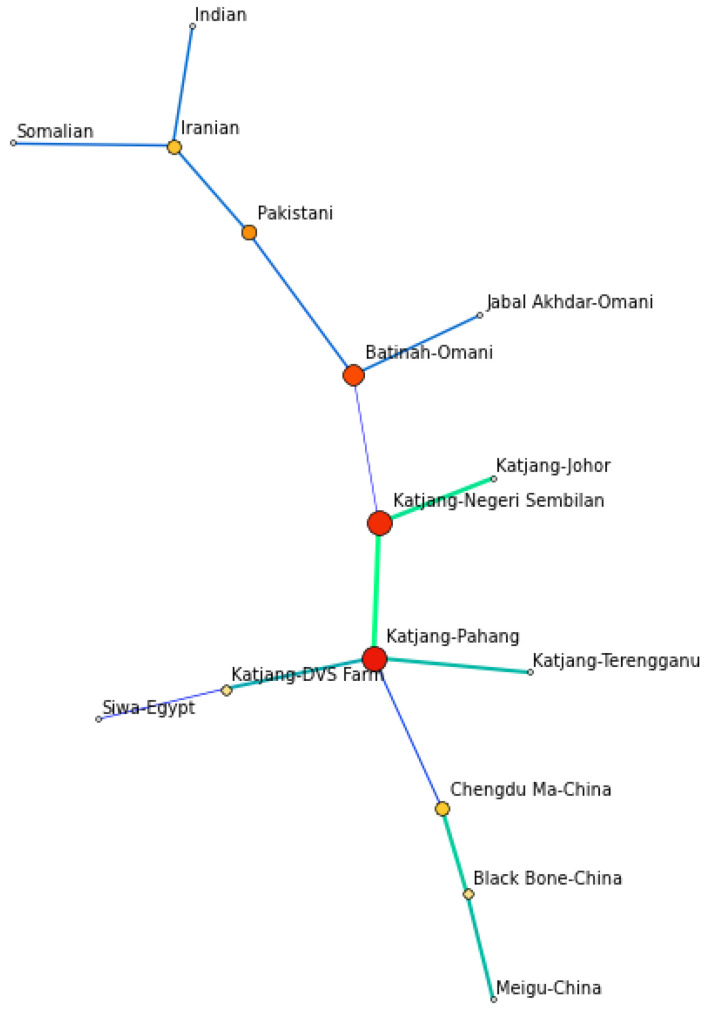
Network analysis among populations based on Goldstein’s genetic distance [52] using EDENetworks [51]. Node sizes are proportionate to the betweenness among populations.

Network analysis was used to assess connectivity of all populations and identify central populations. Figure 5 shows network analysis based on Goldstein’s genetic distance [52]. Katjang goats from Pahang formed the centre of the network and had high betweenness with others. Katjang goats from the DVS Farm are also connected to the Egyptian population. The Terengganu population is interconnected only with the Pahang population. Goats from all other populations from various countries may have the shortest path of connectivity through Katjang of Pahang.

## 4. Discussion

### 4.1. Microsatellite Markers Suitability

Microsatellite polymorphism assessment is useful to evaluate the suitability of the microsatellite panel chosen for the diversity analysis, such as heterozygosity, inbreeding estimates and genetic distances between different populations. The allele diversity obtained reflected the differences in DNA sequences and consequently reflected the genetic diversity [56]. From the 36 microsatellite loci studied in the indigenous Katjang goat breed in Malaysia, one locus (BM6444) showed the absence of alleles from the microsatellite locus amplification, showing the possibility of the occurrence of a null allele at that locus. Null alleles can be present due to high mutation rates in the flanking sequences of the locus [57,58]. 

Of the other 35 microsatellite loci, four loci were found to be monomorphic in this study. Three of the monomorphic loci (INRABERN172, ILTS005, MAF209) were among the list of polymorphic microsatellite loci for goat studies suggested by the FAO [26]. INRABERN172 and ILSTS005 both generated nine alleles and six alleles, as studied in the indigenous Gaddi goat breed from the Western Himalayas [59], while MAF209 produced nine alleles for the indigenous goats of Sub-Saharan Africa [60]. Another locus, RM004, generated 10 alleles in Asian goats, as studied by Barker et al. [6], and also produced nine alleles, as studied in the Berari goat breed from India [61]. The monomorphic microsatellite loci obtained from this study could be due to alleles that had become fixed in the Katjang goat, indicating no genetic variation at these loci. This could be verified by adding more samples from other populations. There is also the possibility that the monomorphic markers observed in this study actually produced alleles that had the same lengths but were not identical in sequence, or homoplasic alleles [62]. To resolve this, sequencing of the alleles should be done instead of genotyping. 

According to Barker [55], each microsatellite marker should contain at least four alleles to reduce the standard error of the distance estimates between populations. However, all these loci generated more than four alleles in other goat studies and had been previously suggested by the International Society of Animal Genetics (ISAG)-FAO working group [63] and endorsed in 2011 for use in the genetic studies of goats [26]. Therefore, the occurrence of the low level of observed alleles in this study might be attributed to other factors, including the occurrence of null alleles, which could affect our genetic diversity estimates of the Katjang goats. Therefore, only 25 polymorphic markers were used for further genetic diversity calculations.

### 4.2. Genetic Diversity of the Katjang Goat

The genetic diversity of the Katjang goats can be assessed by the estimation of heterozygosity, which is the average proportion of individuals that exhibit heterozygous alleles in a population [64]. The Katjang goat was found to generate a mean number of observed heterozygosity (0.29 ± 0.14), which was lower than the effective heterozygosity (0.72 ± 0.14). The observed heterozygosity was found to be lower than previously reported in 2013 for the Katjang goat breed and also lower than other studies in goat genetic diversity [43,45,54,59,61,65,66,67,68,69,70,71,72,73]. This showed that the genetic diversity of Katjang goats is decreasing and contains many loci with homozygous alleles as compared to other goat breeds. The high level of homozygosity could be influenced by multiple factors, such as inbreeding, small population size and demographic history [74]. Measures of heterozygote deficiency through the inbreeding coefficient (F_IS_) suggested that this breed had high inbreeding, with the value of 0.46, which manifested in the decrease in the total number of heterozygous genotypes or an increase in the number of homozygous genotypes. This may seem to suggest that the Katjang goat breed has suffered genetic erosion due to indiscriminate crossbreeding and has been left with little genetic variation. In this study, purebred individuals could only be obtained from farms maintained by small-scale farmers, who used the purebreds only for personal use or sold them in niche markets. Therefore, there was minimum immigration and emigration occurring between farms and between populations.

Bottleneck analysis showed that significant heterozygosity excess was historically found in this breed; this suggests population size reduction has occurred. The test method used in this study exploits the fact that rare alleles are reduced faster than heterozygosity at the mutation-drift equilibrium during a bottleneck event [75]. However, the test of the mode-shift distribution using the allele frequency data [42] produced a normal L-shaped distribution in mode shift, which suggested that a bottleneck was not likely to have occurred recently. According to [75], the test of heterozygosity excess can detect the occurrence of bottlenecks from 25 to 250 generations following the population reduction, while the test of mode-shift distribution can only detect the occurrence of bottlenecks 40–80 generations following the population reduction [42]. In the absence of detailed past information on this breed in Malaysia, it is difficult to identify precisely which factors led to the bottleneck event.

Even though the DVS Farm’s conservation herd has been subjected to inbreeding in the past decade, with no new individuals being brought into the herd to infuse genetic variation, the farm still contains the highest genetic variation as compared to the other populations. This might be attributed to the mixed original geographical distribution of the founder group of individuals initially incorporated into the herd. However, the value of this herd’s heterozygosity was still considered low. Populations from the other four states also contained low genetic variations, even though the individuals were sampled from various farms in each state.

### 4.3. Population Genetic Relationship and Structure

As revealed by the dendogram tree of genetic distance, the Katjang goat population from Pahang had the closest distance to Negeri Sembilan, and they showed a high level of betweenness with each other in the network analysis; thus, it is assumed they had the most amount of gene flow from all populations studied. Notably, almost all Katjang populations grouped together in the MDS plot, except for Terengganu, which can be assumed to be due to its lowest heterozygosity amongst all populations. The connectivity of the network, which was represented by the flow of genes, suggested that Katjang goats have substantial gene flow among all populations studied. However, since this breed is mostly reared by small-scale farmers, thus assuming limited gene flow in current time, it can be suggested that the Katjang goats in Malaysia were historically admixed with various populations from other countries, which influences their genetic make-up. Historical literature suggests that the Katjang breed might have entered Malaysia from India through the Mediterranean, Red Sea and Nile Delta [3] as well as east of Malaysia during ancient times and also possibly via China routes [6]. 

This finding also highlights that, through assessment of genetic structure and connectivity measures that may indicate gene flow among populations, there may not be positively correlation with genetic diversity. The population that emerged as the centroid of the multidimensional scaling, along with centrality of the network, might still have the lowest genetic diversity. Comparatively, even though the Katjang population showed higher betweenness in the network system, the Katjang goat exhibits the lowest heterozygosity from other studied populations [43,45,54]. As the only indigenous breed in Malaysia, having low heterozygosity may deplete the genetic source of this breed, which has been proven to adapt suitably to the local environment and may act as a genetic reservoir for other possible challenges in the future. 

Through measures of genetic relationship and structure, the Katjang goat showed the highest similarity with China, which, in accordance with the previous study of microsatellite and protein loci, suggested that there was possible dispersion of this breed from Western Asia via China [6]. However, even though historical literature has suggested that this breed might have originated from India through morphological features [3], genetic similarity of this breed with the Indian population cannot be proven in this study. To further resolve this, a comprehensive study to assess the genetic relationship of the Katjang goat with the local Indian breeds can be done to validate their genetic similarity and connectivity as well as to provide insight into their migration history.

## 5. Conclusions

This study provides insight into the genetic diversity of the purebred Katjang goat in Malaysia as well as into genetic relationships and structures. The indigenous Katjang goat in Malaysia has low genetic variation as compared to other breeds reported in various goat studies outside Malaysia. Bottleneck events might have occurred from 25 to 250 generations ago, although there was no detailed historical event that could give insight into the reduction in numbers of this breed. This breed also suffered high inbreeding as indicated by F_IS_ (inbreeding coefficient). This result might be influenced by the low availability of the purebred samples, since almost all the samples were obtained from small-scale farmers, and there was minimum genetic drift to permit exchange of genetic variation. This research also proved that a conservation herd set up with the injection of individuals from various geographical areas could lead to success in increasing the genetic variation of the population, even though the population might later go through closed breeding. 

Through the study of population structure, it is suggested that this breed shared substantial gene flow with other breeds from various countries in the past, possibly during ancient migration of this breed into Malaysia. It is also suggested that the genetic make-up of this breed is molded by its ancient spatial originality, and it might have gone through admixture during migration and possible dispersion in the past. Although Katjang goats form the centre of the goat population network analysis and centroid of the multidimensional scaling plot, the genetic diversity of this breed is comparatively low.

Since this breed is the only indigenous domesticated goat in Malaysia and has proven to adapt suitably to the local environment, having low genetic diversity is alarming and might cause possible challenges in the future if this breed is not properly maintained and conserved. In addition, with the indiscriminate practice of crossbreeding and the unknown conservation status of this breed in Malaysia, sustainable conservation is necessary to prevent this breed from further genetic erosion. Hopefully, the characterization of this breed will facilitate the conservation, improvement and maintenance of this Malaysian national asset.

## Figures and Tables

**Table 1:** Details of goat data extracted from published literature.

Goat Population	Origin	Data Numbers	References
Chengdu Ma breed	Chengdu, Sichuan, China	30	[43]
Meigu breed	Meigu, Sichuan, China	34	[43]
Black-bone breed	Wuhan, Hubei, China	24	[43]
Siwa, Egypt	Siwa, Egypt	20	[45]
Jabal Akhdar breed	Oman	31	[54]
Batinah breed	Oman	30	[54]
Somalian	Hargeisa, Somalia	28	[54]
Iranian	Bandar Abbas, Iran	21	[54]
Pakistani	Gwadar, Pakistan	26	[54]
Indian	Malegaon, Nasik, India	21	[54]

**Table 2:** Shared microsatellite loci used for combined analysis of inter-population genetic relationship and structure of Katjang goats with other breeds/populations from published literature.

Breed/ Population	Microsatellite Loci										
	SRCRSP5	MAF065	MAF70	OarFCB48	SRCRSP9	SPS113	OarFCB20	CSRD247	ILSTS029	SRCRSP8	OarAE54
Katjang	X	X	X	X	X	X	X	X	X	X	X
Chengdu Ma	X	X	X	X	X	X	X	X	X	X	X
Meigu	X	X	X	X	X	X	X	X	X	X	X
Black-bone	X	X	X	X	X	X	X	X	X	X	X
Siwa	X		X	X	X	X			X		X
Jabal Akhdar	X	X					X	X		X	
Batinah	X	X					X	X		X	
Somalian	X	X					X	X		X	
Iranian	X	X					X	X		X	
Pakistani	X	X					X	X		X	
Indian	X	X					X	X		X	

“X” indicates data used for combined analysis.

**Table 3:** Information on the 36 microsatellite loci studied.

No	Microsatellite Loci	Loci Reference	Allele Size Range (Base Pair)	Allele Polymorphism
1.	SRCRSP5	[26]	162–185	Polymorphic
2.	MAF065	[26]	119–144	Polymorphic
3.	MAF70	[26]	137–172	Polymorphic
4.	SRCRSP23	[26]	86–107	Less than 4 alleles
5.	OarFCB48	[26]	148–181	Polymorphic
6.	INRA023	[26]	210–219	Less than 4 allele
7.	SRCRSP9	[26]	112–140	Polymorphic
8.	SPS113	[26]	134–157	Polymorphic
9.	INRABERN172	[26]	247	Monomorphic
10.	OarFCB20	[26]	90–112	Polymorphic
11.	CSRD247	[26]	210–273	Polymorphic
12.	MCM527	[26]	154–165	Less than 4 alleles
13.	ILSTS087	[26]	144–165	Polymorphic
14.	INRA063	[26]	174–184	Less than 4 alleles
15.	ILSTS011	[26]	241–297	Polymorphic
16.	ILSTS005	[26]	180	Monomorphic
17.	SRCRSP15	[26]	180–208	Polymorphic
18.	SRCRSP3	[26]	107–132	Polymorphic
19.	ILSTS029	[26]	156–192	Polymorphic
20.	TGLA53	[26]	127–160	Polymorphic
21.	ETH10	[26]	202–212	Less than 4 alleles
22.	MAF209	[26]	109	Monomorphic
23.	INRABERN185	[26]	247–291	Polymorphic
24.	P19(DYA)	[26]	160–195	Polymorphic
25.	TCRVB6	[26]	231–258	Polymorphic
26.	SRCRSP7	[26]	125–135	Less than 4 alleles
27.	SRCRSP8	[26]	209–243	Polymorphic
28.	DRBP1	[26]	107–146	Polymorphic
29.	OarAE54	[26]	114–141	Polymorphic
30.	BM6444	[26]	-	Non-amplification
31.	RM004	[6]	114	Monomorphic
32.	ILSTS044	[30]	160–172	Polymorphic
33.	TGLA245	[6]	125–162	Polymorphic
34.	BM1818	[29]	251–290	Polymorphic
35.	OarJMP29	[31]	123–138	Polymorphic
36.	INRA005	[6]	130–162	Polymorphic

**Table 4:** Genetic diversity obtained across the five populations of the Katjang goat breed based on 25 microsatellite markers.

No	Microsatellite Loci	^1^ Na	^2^ Ne	^3^ Ho	^4^ He	^5^ F_IS_
1.	SRCRSP5	6	4.19	0.33	0.77	0.44
2.	MAF065	6	5.73	0.59	0.83	0.11
3.	MAF70	8	4.26	0.44	0.77	0.39
4.	OarFCB48	8	3.80	0.38	0.74	0.42
5.	SRCRSP9	8	5.87	0.41	0.83	0.34
6.	SPS113	6	3.10	0.22	0.68	0.65
7.	OarFCB20	5	2.26	0.12	0.56	0.64
8.	CSRD247	15	10.05	0.46	0.91	0.34
9.	ILSTS087	6	4.84	0.34	0.80	0.32
10.	ILSTS011	9	4.26	0.31	0.77	0.39
11.	SRCRSP15	5	3.51	0.21	0.72	0.57
12.	SRCRSP3	7	5.41	0.47	0.82	0.26
13.	ILSTS029	8	1.69	0.17	0.41	0.52
14.	TGLA53	7	2.82	0.38	0.65	0.33
15.	INRABERN185	6	1.37	0.16	0.27	0.51
16.	P19(DYA)	6	2.70	0.48	0.63	−0.06
17.	TCRVB6	8	4.95	0.08	0.80	0.80
18.	SRCRSP8	8	5.49	0.08	0.82	0.88
19.	DRBP1	10	5.54	0.15	0.83	0.79
20.	OarAE54	8	3.92	0.28	0.75	0.49
21.	ILSTS044	4	2.56	0.19	0.62	0.37
22.	TGLA245	8	4.54	0.19	0.79	0.64
23.	BM1818	9	5.78	0.28	0.83	0.53
24.	OarJMP29	4	2.48	0.28	0.60	0.31
25.	INRA005	6	3.90	0.31	0.75	0.37
	Mean	7.24	4.20	0.29	0.72	0.46
	Standard deviation	2.24	1.80	0.14	0.14	

^1^ Number of allele; ^2^ effective number of alleles; ^3^ observed heterozygosity; ^4^ expected heterozygosity; ^5^ inbreeding coefficient.

**Table 5:** Genetic diversity parameters based on five populations of the Katjang goat breed.

Population	^1^ Ho	^2^ He	^3^ F_IS_
DVS Farm	0.37 ± 0.23	0.52 ± 0.20	0.25
Negeri Sembilan	0.28 ± 0.19	0.63 ± 0.18	0.55
Pahang	0.29 ± 0.16	0.66 ± 0.17	0.56
Johor	0.30 ± 0.21	0.54 ± 0.19	0.40
Terengganu	0.25 ± 0.24	0.51 ± 0.26	0.48

^1^ Observed heterozygosity; ^2^ expected heterozygosity; ^3^ inbreeding coefficient.

## Data Availability

The data presented in this study are available upon request from the corresponding author.

## References

[1] Department of Veterinary Services Malaysia Malaysia: Perangkaan Ternakan. http://www.dvs.gov.my/dvs/resources/user_1/2020/BP/Perangkaan/3.Muka_Surat_1-12_OK_.pdf.

[2] Department of Veterinary Services Malaysia Malaysia: Perangkaan Ternakan Import. http://www.dvs.gov.my/dvs/resources/user_1/2020/BP/Perangkaan/2._Malaysia_Import_.pdf.

[3] Devendra C. (1966). Goat Breeds of Malaysia. Malays. Agric. J..

[4] Devendra C., Nozawa K. (1976). Goats in South East Asia-Their Status and Production. Z. Tierz. Züchtungsbiol..

[5] Devendra C. (2007). Goats: Biology, Production, and Development in Asia.

[6] Barker J.S.F., Tan S.G., Moore S.S., Mukherjee T.K., Matheson J.L., Selvaraj O.S. (2001). Genetic Variation within and Relationships Among Populations of Asian Goats (Capra Hircus). J. Anim. Breed. Genet..

[7] Solaiman S.G. (2010). Goat Science and Production.

[8] Alimon A.R. (1988). Penternakan Kambing.

[9] Department of Veterinary Services Malaysia (2003). First Report on the State of the World’s Animal Genetic Resources: Animal Genetic Resources in Malaysia.

[10] Devendra C. (1966). The Importance of Goats in Malaya. J. Anim. Breed. Genet..

[11] Devendra C., McLeroy G.B. (1982). Goat and Sheep Production in the Tropics.

[12] Devendra C. (1966). Studies in the Nutrition of the Indigenous Goat of Malaya. 1. The Body Measurements, Composition of Sample Joints and Their Relationship to Carcass Composition. Malays. Agric. J..

[13] Devendra C. (1986). Pemeliharaan dan Pengeluaran Kambing.

[14] Devendra C., Burns M. (1970). Goat production in the tropics. Technical Communications. Commonwealth Bureau of Animal Breeding and Genetics.

[15] Peters K., Deichert G., Drewes E., Fichtner G., Moll S., Chavarria F., Diakite B. (1979). Goat Production in Low Income Economic Units of Selected Areas in West-Malaysia. Proceedings of the Schriften des Seminars fuer Landwirtschaftliche Entwicklung.

[16] Gosling L.A.P. (1958). Patterns and Problems of Livestock Production in Malaya. Ph.D. Thesis.

[17] Hirooka H., Mukherjee T.K., Panandam J.M., Horst P. (1997). Genetic Parameters for Growth Performance of the Malaysian Local Goats and Their Crossbreds with the German (Improved) Fawn Goats. J. Anim. Breed. Genet..

[18] Porter V., Alderson L., Hall S.J.G., Sponenberg D.P. (2016). Mason’s World Encyclopedia of Livestock Breeds and Breeding.

[19] Ariff O.M., Hifzan R.M., Zuki A.B.M., Jiken A.J., Lehan S.M. (2010). Maturing Pattern for Body Weight, Body Length and Height at Withers of Jamnapari and Boer Goats. Pertanika J. Trop. Agric. Sci..

[20] Hifzan M.R., Nor Amna A.M.N., Izuan Bahtiar A.J.B., Amie Marini A.B., Mohd Hafiz A.W. Manipulating of Katjang Goat Genetic Material for Sustainable Goat Industry in Malays. http://ap.fftc.agnet.org/ap_db.php?id=950&print=1.

[21] Tsukahara Y., Chomei Y., Oishi K., Kahi A.K., Panandam J.M., Mukherjee T.K., Hirooka H. (2008). Analysis of Growth Patterns in Purebred Kambing Katjang Goat and its Crosses with the German Fawn. Small Rumin. Res..

[22] Department of Veterinary Services Malaysia, Livestock Breeding Policy Malaysian Livestock Breeding Policy 2013. http://www.dvs.gov.my/dvs/resources/user_1/DVSpdf/Livestock_Breeding_Policy.pdf.

[23] DAD-IS Domestic Animal Diversity Information System (DAD-IS). http://www.fao.org/dad-is/browse-by-country-and-species/en/.

[24] FAO (2013). In Vivo Conservation of Animal Genetic Resources.

[25] Marwal A., Sahu A.K., Gaur R.K., Verma A., Singh A. (2014). Molecular Markers: Tool for Genetic Analysis. Animal Biotechnology Models in Discovery and Translation.

[26] FAO (2011). Molecular Genetic Characterization of Animal Genetic Resources.

[27] Li Y.C., Korol A.B., Fahima T., Beiles A., Nevo E. (2002). Microsatellites: Genomic Distribution, Putative Functions and Mutational Mechanisms: A Review. Mol. Ecol..

[28] Marini A., Hifzan M. (2013). Genetic Variation of Four Goat Breeds in Malaysia Using Microsatellite Polymorphism Markers. Malays. J. Anim. Sci..

[29] Li M.H., Zhao S.H., Bian C., Wang H.S., Wei H., Liu B., Yu M., Fan B., Chen S.L., Zhu M.J. (2002). Genetic Relationships Among Twelve Chinese Indigenous Goat Populations Based on Microsatellite Analysis. Genet. Sel. Evol..

[30] Kemp S.J., Brezinsky L., Teale A.J. (1993). A Panel of Bovine, Ovine and Caprine Polymorphic Microsatellites. Anim. Genet..

[31] Balasingham T.G., Robinson N.A., McGregor B.A. (1999). Implications for the Conservation of Genetic Diversity in Mohair Goats from a Comparison of a Relic Island Population with Breeds Farmed in Australia. Aust. J. Exp. Agric..

[32] Lazar I., Lazar I. Gel Analyzer 2010: Freeware 1D gel Electrophoresis Image Analysis Software. http://www.gelanalyzer.com.

[33] Glaubitz J.C. (2004). CONVERT: A User-Friendly Program to Reformat Diploid Genotypic Data for Commonly used Population Genetic Software Packages. Mol. Ecol. Notes.

[34] Yeh F., Boyle T. (1997). Population Genetic Analysis of Co-Dominant and Dominant Markers and Quantitative Traits. Belg. J. Bot..

[35] Kimura M., Crow J.F. (1964). The Number of Alleles that can be Maintained in a Finite Population. Genetics.

[36] Levene H. (1949). On a Matching Problem Arising in Genetics. Ann. Math. Stat..

[37] Stern C. (1943). The Hardy-Weinberg Law. Science.

[38] Nei M. (1987). Molecular Evolutionary Genetics.

[39] Piry S., Luikart G., Cornuet J.M. (1999). Bottleneck: A Computer Program for Detecting Recent Reductions in the Effective Population Size Using Allele Frequency Data. J. Hered..

[40] Di Rienzo A., Peterson A.C., Garza J.C., Valdes A.M., Slatkin M., Freimer N.B. (1994). Mutational Processes of Simple-Sequence Repeat Loci in Human Populations. Proc. Natl. Acad. Sci. USA.

[41] Wilcoxon F. (1945). Individual Comparisons by Ranking Methods. Biom. Bull..

[42] Luikart G., Allendorf F.W., Cornuet J.M., Sherwin W.B. (1998). Distortion of Allele Frequency Distributions Provides a Test for Recent Population Bottlenecks. J. Hered..

[43] Guang-Xin E., Zhao Y.J., Chen L.P., Ma Y.H., Chu M.X., Li X.L., Hong Q.H., Li L.H., Guo J.J., Zhu L. (2018). Genetic Diversity of the Chinese Goat in the Littoral Zone of the Yangtze River as Assessed by Microsatellite and mtDNA. Ecol. Evol..

[44] Al-Araimi N.A., Gaafar O.M., Costa V., Neira A.L., Al-Atiyat R.M., Beja-Pereira A. (2017). Genetic Origin of Goat Populations in Oman Revealed by Mitochondrial DNA Analysis. PLoS ONE.

[45] El-Sayed M., Al-Soudy A., El Badawy A. (2016). Microsatellite Markers Polymorphism between Two Egyptian Goat Populations (Capra Hircus). Egypt. J. Genet. Cytol..

[46] Nei M., Tajima F., Tateno Y. (1983). Accuracy of Estimated Phylogenetic Trees from Molecular Data. J. Mol. Evol. Evol..

[47] Takezaki N., Nei M., Tamura K. (2010). POPTREE2: Software for Constructing Population Trees from Allele Frequency Data and Computing other Population Statistics with Windows Interface. Mol. Biol. Evol..

[48] Pena J., Gómez-Pérez L., Alfonso-Sánchez M. (2020). GenoCline: On the Trail of Spatial Patterns of Genetic Variation. Authorea Prepr..

[49] Kruskal J.B. (1964). Nonmetric Multidimensional Scaling: A Numerical Method. Psychometrika.

[50] Reynolds J., Weir B.S., Cockerham C.C. (1983). Estimation of the Coancestry Coefficient: Basis for a Short-Term Genetic Distance. Genetics.

[51] Kivelä M., Arnaud-Haond S., Saramäki J. (2015). EDENetworks: A User-Friendly Software to Build and Analyse Networks in Biogeography, Ecology and Population Genetics. Mol. Ecol. Resour..

[52] Goldstein D.B., Ruiz Linares A., Cavalli-Sforza L.L., Feldman M.W. (1995). Genetic Absolute Dating Based on Microsatellites and the Origin of Modern Humans. Proc. Natl. Acad. Sci. USA.

[53] Stauffer D., Aharony A. (1994). Introduction to Percolation Theory.

[54] Al-Araimi N.A., Al-Atiyat R.M., Luzuriaga-Neira A., Mahgoub Gaafar O., Kadim I.T., Al-Marzooqi W., Babiker H.A., Al-Kindi M.N., Al-Ansari A.S., Al-Lawati A.H. (2019). Genetic Structure of Omani Goats Reveals Admixture Among Populations from Geographically Proximal Sites. Small Rumin. Res..

[55] Barker J.S.F. A Global Protocol for Determining Genetic Distances Among Domestic Livestock Breeds. Proceedings of the 5th World Congress on Genetics Applied to Livestock Production.

[56] FAO (2007). The State of The World’s Animal Genetic Resources for Food and Agriculture.

[57] Chapuis M.P., Estoup A. (2007). Microsatellite Null Alleles and Estimation of Population Differentiation. Mol. Biol. Evol..

[58] Callen D.F., Thompson A.D., Shen Y., Phillips H.A., Richards R.I., Mulley J.C., Sutherland G.R. (1993). Incidence and Origin of “Null” Alleles in the (AC)n Microsatellite Markers. Am. J. Hum. Genet..

[59] Singh G., Thakur Y., Kour A., Sankhyan V., Katoch S. (2015). Genetic Characterization of Gaddi Goat Breed of Western Himalayas Using Microsatellite Markers. Vet. World.

[60] Hirbo J., Kemp S.J., Hanotte O., Kifaro G.C., Watts P.C., Gwakisa P.S., Petersen P.H., Chenyambuga S.W., Rege J.E.O. (2014). Genetic Characterization of Indigenous Goats of Sub-Saharan Africa Using Microsatellite DNA Markers. Asian-Australas. J. Anim. Sci..

[61] Kharkar K., Kuralkar S.V., Kuralkar P. (2015). Molecular Genetic Characterization of Berari Breed of Goat Using Microsatellite Markers. Indian J. Anim. Res..

[62] Jarne P., Lagoda P.J.L. (1996). Microsatellites, from Molecules to Populations and Back. Trends Ecol. Evol..

[63] ISAG/FAO (2004). Secondary Guidelines for Development of National Farm Animal Genetic Resources Management Plans. Measurement of Domestic Animal Diversity (MoDAD): Recommended Microsatellite Markers.

[64] Hanotte O., Jianlin H. (2005). Genetic Characterization of Livestock Populations and its Use in Conservation Decision Making. Role Biotechnol. Explor. Prot. Genet. Resour..

[65] Martínez A.M., Carrera M.P., Acosta J.M., Rodríguez-Gallardo P.P., Cabello A., Camacho E., Delgado J.V. (2004). Genetic Characterisation of the Blanca Andaluza Goat Based on Microsatellite Markers. S. Afr. J. Anim. Sci..

[66] Álvarez I., Traoré A., Kaboré A., Zaré Y., Fernández I., Tamboura H.H., Goyache F. (2012). Microsatellite Analysis of the Rousse de Maradi (Red Sokoto) goat of Burkina Faso. Small Rumin. Res..

[67] Li X.L., Valentini A. (2004). Genetic Diversity of Chinese Indigenous Goat Breeds Based on Microsatellite Markers. J. Anim. Breed. Genet..

[68] Tefiel H., Ata N., Chahbar M., Benyarou M., Fantazi K., Yilmaz O., Cemal I., Karaca O., Boudouma D., Gaouar S.B.S. (2018). Genetic Characterization of four Algerian Goat Breeds Assessed by Microsatellite Markers. Small Rumin. Res..

[69] Korkmaz Aĝaoĝlu Ö., Ertuĝrul O. (2012). Assessment of Genetic Diversity, Genetic Relationship and Bottleneck Using Microsatellites in Some Native Turkish Goat Breeds. Small Rumin. Res..

[70] Naqvi A.N., Bukhari J.F., Vahidi S.M.F., Utsunomiya Y.T., Garcia J.F., Babar M.E., Han J.L., Pichler R., Periasamy K. (2017). Microsatellite Based Genetic Diversity and Mitochondrial DNA D-Loop Variation in Economically Important Goat Breeds of Pakistan. Small Rumin. Res..

[71] Al-Atiyat R.M., Alobre M.M., Aljumaah R.S., Alshaikh M.A. (2015). Microsatellite Based Genetic Diversity and Population Structure of Three Saudi Goat Breeds. Small Rumin. Res..

[72] Negrini R., D’Andrea M., Crepaldi P., Colli L., Nicoloso L., Guastella A.M., Sechi T., Bordonaro S., Ajmone-Marsan P., Pilla F. (2012). Effect of Microsatellite Outliers on the Genetic Structure of Eight Italian Goat Breeds. Small Rumin. Res..

[73] Awobajo O.K., Salako A.E., Osaiyuwu O.H. (2015). Analysis of Genetic Structure of Nigerian West African Dwarf Goats by Microsatellite Markers. Small Rumin. Res..

[74] Cardoso T.F., Amills M., Bertolini F., Rothschild M., Marras G., Boink G., Jordana J., Capote J., Carolan S., Hallsson J.H. (2018). Patterns of Homozygosity in Insular and Continental Goat Breeds. Genet. Sel. Evol..

[75] Cornuet J.M., Luikart G. (1996). Description and Power Analysis of Two Tests for Detecting Recent Population Bottlenecks from Allele Frequency Data. Genetics.

